# Multifunctional Hydrogel Dressing Loaded with DH-EVs Promotes Healing of Bacteria-Infected Diabetic Wounds

**DOI:** 10.3390/nano16181171

**Published:** 2026-09-16

**Authors:** Jiemin Dai, Wenwen Zhao, Xin Ma, Wantong Lu, Wentao Zhou, Kan Yin, Meng Zhao

**Affiliations:** 1 School of Nursing, Qingdao University, Qingdao 266071, China; 2 School of Pharmacy, Qingdao University, Qingdao 266071, China; 3 Qingdao Walson Standard Biopharmaceutical Co., Ltd., Qingdao 266000, China

**Keywords:** Chinese herbal medicine-derived extracellular vesicle-like particles, diabetic wound, hydrogel, wound dressing

## Abstract

**Background:** Persistent inflammation and excessive neutrophil extracellular trap (NET) formation hinder the healing of bacteria-infected diabetic wounds. The *Salvia miltiorrhiza*–*Astragalus membranaceus* herb pair possesses anti-inflammatory activity, but its poor solubility, rapid metabolic clearance, and unclear material basis limit direct wound application. Extracellular vesicles (DH-EVs) isolated from this herb pair may improve local delivery and stability. Therefore, this work constructs a DH-EV-incorporated hydrogel dressing aimed at inhibiting NET formation and facilitating diabetic wound healing. **Methods:** DH-EVs were isolated from the *Salvia miltiorrhiza*–*Astragalus membranaceus* pair, characterized by lipidomics and small-RNA sequencing, and incorporated into a hydrogel. The dressing was evaluated for physicochemical properties, mechanical performance, biocompatibility, NET inhibition, anti-inflammatory activity, and therapeutic efficacy in vitro and in bacteria-infected diabetic wounds. **Results:** DH-EVs were isolated, and sequencing identified miRNAs potentially regulating inflammation- and NET-related pathways. Incorporation into hydrogel did not significantly alter mechanical integrity or biocompatibility. In vitro and in vivo, the DH-EV-loaded hydrogel suppressed excessive NET formation, reduced local inflammation, and improved wound closure and tissue regeneration in bacteria-infected diabetic wounds. **Conclusions:** The DH-EV-loaded hydrogel promotes bacteria-infected diabetic wound repair by suppressing excessive NET formation and inflammation, providing a promising strategy for localized wound treatment.

## 1. Introduction

Diabetes mellitus is a metabolic disorder characterized by chronic hyperglycemia and accompanied by long-term typical complications [1]. Diabetic wounds are one of its most common complications, not only reducing patients’ quality of life and imposing substantial economic and psychological burdens, but also carrying high rates of recurrence and amputation [2,3]. Delayed healing of diabetic wounds is influenced by multiple factors, including hyperglycemia, oxidative stress, vascular neuropathy, and chronic inflammation, among which chronic inflammation is considered a critical factor in impaired wound healing and is closely associated with immune cell dysfunction [4].

Compared with normal wounds, diabetic wounds are continuously bathed in a hyperglycemic milieu, which facilitates bacterial invasion into wound tissues and cellular proliferation, while promoting the secretion of extracellular polymeric substances to form biofilms, thereby leading to infection of diabetic wounds [5]. When persistent bacterial infection occurs in diabetic wounds, the inflammatory response is amplified, further prolonging the inflammatory phase and preventing normal transition to the proliferative phase, ultimately exacerbating wound healing delay [6]. Our model breaks through the limitations of traditional single-factor studies by simulating the complex interplay between the diabetic hyperglycemic environment and bacterial infection, accurately recapitulating the chronic wound microenvironment (e.g., persistent inflammation, oxidative stress, etc.). Macrophage polarization has long been considered a key factor in diabetic wound inflammation; however, with advancing research on bacteria-infected diabetic wounds, neutrophil extracellular traps (NETs) have also been confirmed to be extensively involved. NETs are reticular structures produced by neutrophils, containing decondensed chromatin and intracellular granule proteins. During normal wound healing, neutrophils secrete small amounts of NETs to effectively clear pathogens, combat bacterial infection, and promote wound healing [7]. Nevertheless, in the bacteria-infected diabetic wound microenvironment, prolonged overactivation and dysregulated apoptosis of neutrophils lead to excessive release of NETs [8], which in turn promotes macrophage polarization toward the M1 phenotype, elevates NLRP3 inflammasome and IL-1β levels, amplifies local inflammatory responses, and impairs wound healing [9]. This microenvironment exhibits a tripartite pathological feature of “infection–inflammation–metabolic disorder,” necessitating multi-target intervention strategies.

*Salvia miltiorrhiza* and *Astragalus membranaceus* are traditional Chinese medicines with anti-inflammatory and antibacterial functions, and have been demonstrated to effectively inhibit NET formation [10,11]. However, *Salvia miltiorrhiza* and *Astragalus membranaceus* suffer from poor solubility, rapid metabolic clearance, and unclear material composition, which limit their application in cutaneous wound healing [12]. Plant-derived extracellular vesicles (PDEVs) are small vesicles released by plant cells that contain bioactive molecules such as proteins, nucleic acids, and lipids, and are currently gaining increasing attention as a novel drug delivery system for traditional Chinese medicine [13]. Compared with conventional traditional Chinese medicines, PDEVs offer superior bioavailability and more clearly defined material composition [14]. Current research often combines PDEVs with appropriate materials to exert their therapeutic effects in disease treatment [5,15].

Hydrogel dressings are novel wound dressings with three-dimensional network structures, exhibiting good biocompatibility and drug-loading capacity, and can effectively encapsulate PDEVs [16]. Among these, hydrogels prepared from oxidized sodium alginate possess favorable breathability and water absorption, making them particularly suitable for bacteria-infected diabetic wounds with increased exudate [17]. Plant-derived extracellular vesicles can retain various bioactive phytochemicals, including flavonoids and saponins, and the associated bioactive components can remain relatively stable following processing or decoction. Meanwhile, the natural phospholipid bilayer structure of PDEVs makes them favorable carriers for the delivery of bioactive components [18,19]. In addition, incorporation of PDEVs into hydrogels can improve local retention and enable sustained release, while PDEV-loaded hydrogels can exhibit favorable mechanical properties [20]. Therefore, this study aimed to construct a sodium alginate-based hydrogel dressing loaded with *Salvia miltiorrhiza*–*Astragalus membranaceus* extracellular vesicles (DH-EVs) to inhibit excessive NET formation in bacteria-infected diabetic wounds and to evaluate its therapeutic effect on diabetic wound healing.

## 2. Materials and Methods

### 2.1. Experimental Materials

Sodium alginate (Cat. No. S100126; viscosity: 200 ± 20 mPa·s) and carboxymethyl chitosan (Cat. No. C304738; carboxylation degree ≥ 80%; viscosity ≤ 100 mPa·s for a 1% aqueous solution at 20 °C) were purchased from Aladdin (Shanghai, China); dopamine hydrochloride from Macklin (Shanghai, China); and the neutrophil isolation kit and PMA from Solarbio (Beijing, China). Anti-Ly-6G and anti-CitH3 antibodies were obtained from ABclonal (Wuhan, China) and Zen-Bioscience (Chengdu, China), respectively. Anti-myeloperoxidase antibody, HRP-conjugated goat anti-rabbit IgG, and FITC-conjugated goat anti-rat IgG were purchased from Boster (Wuhan, China). The mouse myeloperoxidase-DNA complex (MPO-DNA) ELISA kit was obtained from Zcibio (Shanghai, China).

### 2.2. Isolation and Culture of Neutrophils

All neutrophils used were isolated from mouse bone marrow utilizing the Mouse Bone Marrow Neutrophil Isolation Kit (Solarbio Science & Technology Co., Ltd., Beijing, China). Mice were euthanized by cervical dislocation, and femurs and tibiae were harvested. Bone marrow was flushed with phosphate-buffered saline (PBS, Solarbio Science & Technology Co., Ltd., Beijing, China), filtered, and neutrophils were isolated using a mouse bone marrow neutrophil isolation kit, followed by subsequent culture in RPMI-1640 medium (Meilunbio, Dalian, China). L929 mouse fibroblast cells (NCTC clone 929; ATCC CCL-1; ATCC, Manassas, VA, USA), maintained as cryopreserved stocks in our laboratory, were thawed before use and subsequently cultured in RPMI-1640 medium. *Staphylococcus aureus* (Gram-positive, ATCC 25923; ATCC, Manassas, VA, USA) was used for the antibacterial experiments.

### 2.3. Extraction and Characterization of DH-EVs

*Salvia miltiorrhiza* and *Astragalus membranaceus* (Tongrentang, Beijing, China) were cleaned of dust and then decocted to obtain a *Salvia miltiorrhiza*–*Astragalus membranaceus* decoction. After filtration, the decoction was subjected to high-speed centrifugation sequentially at 1000× *g*, 2000× *g*, 3000× *g*, and 10,000× *g* at 4 °C (JXN-26, Beckman Coulter, Brea, CA, USA) to remove impurities. The resulting supernatant was centrifuged at 150,000× *g* for 90 min at 4 °C (Himac CS 120FNX, Hitachi, Tokyo, Japan), and the obtained pellet was resuspended in PBS and stored at −80 °C.

The morphological characteristics of DH-EVs were characterized using transmission electron microscopy (TEM; HT7700, Hitachi, Tokyo, Japan). Particle size and zeta potential were measured by nanoparticle tracking analysis (NTA; ZetaView PMX-120, Particle Metrix, Meerbusch, Germany). Total protein content was determined using the bicinchoninic acid (BCA; MeilunBio, Dalian, China) method. Additionally, lipid and miRNA sequencing of DH-EVs was performed.

### 2.4. Preparation of Multifunctional Hydrogel Loaded with DH-EVs

Oxidized sodium alginate (OSA) was prepared by periodate oxidation of sodium alginate. Briefly, sodium alginate was dissolved in deionized water to obtain a 2.5% (*w*/*v*) solution and magnetically stirred for 12 h until completely dissolved. The pH of the solution was adjusted to 5.0 using HCl (Sinopharm Chemical Reagent Co., Ltd., Shanghai, China). Sodium periodate (NaIO_4_; Macklin, Shanghai, China), dissolved in deionized water, was then added at a NaIO_4_-to-sodium alginate repeating-unit molar ratio of 0.60, corresponding to a theoretical oxidation degree of 60%. The oxidation reaction was carried out under magnetic stirring in the dark for 6 h. Ethylene glycol (Sinopharm Chemical Reagent Co., Ltd., Shanghai, China) was subsequently added to terminate the reaction, followed by stirring for an additional 2 h. The resulting solution was dialyzed against deionized water using dialysis tubing with a molecular-weight cutoff (MWCO) of 3500 Da for 5 days. After dialysis, the purified OSA solution was frozen and lyophilized for 3 days (Alpha 1-2 LDplus, Martin Christ, Osterode am Harz, Germany).

For the synthesis of dopamine-grafted OSA (DA-OSA), lyophilized OSA was dissolved in 50 mmol/L MES buffer (MES, Aladdin, Shanghai, China) (pH 6.0) at a concentration of 5 mg/mL and stirred for 2 h. N-Hydroxy succinimide (NHS) and 1-ethyl-3-(3-dimethylaminopropyl) carbodiimide hydrochloride (EDC·HCl; Aladdin, Shanghai, China) were subsequently added to final concentrations of approximately 25 mmol/L each, and the mixture was allowed to react at room temperature for 30 min to activate the carboxyl groups of OSA. Dopamine hydrochloride was then added at a concentration of approximately 53 mmol/L, and the pH was adjusted to 5.0. The reaction was continued under stirring at room temperature for 24 h. The resulting product was dialyzed against deionized water using dialysis tubing with an MWCO of 3500 Da for 5 days, with the dialysis water replaced 4–5 times. The purified product was subsequently frozen and lyophilized for 3 days to obtain DA-OSA.

For hydrogel preparation, DA-OSA and carboxymethyl chitosan (CMC) were separately dissolved in PBS (pH 7.4) at concentrations of 12% (*w*/*v*) and 5% (*w*/*v*), respectively. The two components were mixed at a DA-OSA/CMC mass ratio of 3:5 (*w*/*w*). The DA-OSA solution was gradually added to the CMC solution in several aliquots, with vortex mixing after each addition until a homogeneous hydrogel was formed.

The hydrogel was mainly formed through dynamic Schiff-base reactions. Periodate oxidation introduced aldehyde groups into sodium alginate to form OSA, while EDC/NHS-mediated conjugation grafted dopamine onto OSA through amide bond formation. During hydrogel formation, the remaining aldehyde groups of DA-OSA reacted with the amino groups of CMC to form dynamic imine (C=N) bonds, thereby contributing to hydrogel formation.

The total protein concentration of the isolated DH-EV preparation was determined using a BCA protein assay. Based on the measured protein concentration, DH-EVs were incorporated at protein-equivalent loading levels of 0%, 2%, or 10% (*w*/*w*) relative to the mass of CMC. The required amount of DH-EV preparation was calculated proportionally for each batch and incorporated into the hydrogel by vortex mixing. The resulting hydrogels were designated DA-OSA-CMC-0%DH, DA-OSA-CMC-2%DH, and DA-OSA-CMC-10%DH, respectively.

### 2.5. Characterization of Multifunctional Hydrogel Loaded with DH-EVs

#### 2.5.1. Fourier Transform Infrared Spectroscopy (FTIR) Analysis

FTIR spectral analysis of lyophilized samples of the three hydrogels was performed using a Fourier transform infrared spectrometer (Nicolet is50, Thermo Fisher Scientific, Waltham, MA, USA) over a scanning range of 4000 cm^−1^ to 500 cm^−1^.

#### 2.5.2. Scanning Electron Microscopy (SEM) Observation and Porosity Analysis

The three types of hydrogels were freeze-dried for 3 days, immersed in liquid nitrogen for 1 min, fractured with tweezers, and their cross-sectional morphology was characterized using scanning electron microscopy (SEM; JSM-IT500, JEOL, Akishima, Japan) at an accelerating voltage of 20 kV. The SEM images obtained at the same magnification were analyzed using ImageJ software (version 1.51k, National Institutes of Health, Bethesda, MD, USA). After grayscale conversion and threshold segmentation, the SEM-derived apparent porosity was calculated as follows:

SEM-derived apparent porosity = (*A*_pore_/*A*_total_) × 100%

The bulk porosity of the hydrogels was further determined using the ethanol displacement method. Freeze-dried hydrogel samples were weighed (*M*_1_), and their diameter and thickness were measured to calculate the sample volume (*V*). The samples were immersed in absolute ethanol (Macklin, Shanghai, China) for 48 h, after which excess ethanol on the surface was removed with filter paper and the samples were immediately reweighed (*M*_2_). Bulk porosity was calculated as follows:

Bulk porosity = [(*M*_2_ − *M*_1_)/(*ρV*)] × 100%

where ρ is the density of absolute ethanol.

#### 2.5.3. Rheological Measurements

The rheological properties of the three hydrogels were evaluated using an advanced rotational rheometer (Malvern Kinexus lab+, Malvern Instruments Ltd., Worcestershire, UK). Oscillatory strain sweep tests were performed on the samples at 25 °C with a fixed frequency of 10 Hz and a strain range of 1–100%.

#### 2.5.4. Swelling Performance Test

Lyophilized samples of the three hydrogels were weighed (*W*_0_) and then placed in PBS solution. At 0.5, 1, 2, 4, 6, 8, 12, 16, and 24 h, samples were removed, surface moisture was blotted dry, and they were reweighed (*W*_t_). Swelling ratio was calculated as:

Swelling ratio = [(*W*_t_ − *W*_0_)/W_0_] × 100%

#### 2.5.5. Degradation Performance Test

Lyophilized samples of the three hydrogels were weighed (*W*_0_) and placed in PBS solution. At 1, 3, 5, and 7 days, samples were removed, rinsed with deionized water to remove residual components on the surface, lyophilized, and reweighed (*W*_t_). Degradation rate was calculated as:

Degradation rate = [(*W*_0_− *W*_t_)/*W*_0_] × 100%

#### 2.5.6. In Vitro DH-EV Release Test

DA-OSA-CMC-2%DH and DA-OSA-CMC-10%DH hydrogels (0.01 g) were immersed in PBS (pH 7.4) and incubated at 37 °C. At 1, 3, 6, 12, 24, 48, and 72 h, the release medium was collected and replaced with an equal volume of fresh PBS. The amount of released DH-EVs was determined using a BCA protein assay at 562 nm (multimode microplate reader, PerkinElmer, Waltham, MA, USA). The cumulative release was calculated as:

Cumulative release = (*M*_t_/*M*_0_) × 100%

#### 2.5.7. Water Vapor Transmission Rate Test

Water vapor transmission rate (WVTR) was determined using a gravimetric method. Each 15 mL centrifuge tube was filled with 5 mL of de-ionized water, and the hydrogel samples were placed over the tube opening and sealed around the edges. The initial mass of the entire assembly was weighed (*M*_0_). After 24 h, the assembly was re-weighed (*M*_t_). The inner diameter of the tube opening was 14.66 mm, corresponding to an effective permeation area (*A*) of 1.688 × 10^−4^ m^2^. WVTR was calculated as:

WVTR =(*M*_0_ − *M*_t_)/(*A* × *t*)

### 2.6. In Vitro Experiments

#### 2.6.1. Preparation of Extracts

The pre-prepared multifunctional hydrogel dressings were first sterilized under ultraviolet irradiation for 12 h, then immersed in RPMI-1640 medium for 24 h. The extracts were filtered through a 0.22 μm filter membrane to remove impurities and stored at 4 °C for later use.

#### 2.6.2. In Vitro Biocompatibility

(1) Hemolysis Assay

Mouse orbital blood was collected and centrifuged at 1000 rpm for 10 min. The supernatant was discarded, and the pellet was washed and centrifuged repeatedly until the supernatant became clear. The erythrocytes were then resuspended in PBS to prepare a 20% erythrocyte suspension. The suspension was incubated with ultrapure water (positive control), PBS (negative control), or hydrogel extracts at 37 °C for 30 min. After centrifugation, photographs were taken, and the supernatant was collected to measure absorbance at 454 nm for hemolysis rate calculation.

(2) Cell Viability Assay

The cytocompatibility of the dressing extracts was evaluated using fibroblasts and neutrophils by a Cell Counting Kit-8 (CCK-8) assay (Beyotime Biotechnology, Shanghai, China). For fibroblast viability assessment, fibroblasts were cultured with extracts from the three types of dressings for 24 h. For neutrophil viability assessment, isolated neutrophils were co-cultured with the corresponding dressing extracts for 4 h. After incubation, cell viability was quantitatively determined using a CCK-8 kit according to the manufacturer’s instructions. Cells cultured without dressing extracts served as the control group. Cell viability was expressed as a percentage relative to that of the control group.

#### 2.6.3. Evaluation of the Antibacterial and Anti-Adhesive Properties

The antibacterial and anti-adhesive properties of the hydrogels against *Staphylococcus aureus* (*S. aureus*) were evaluated using a plate-counting assay. For the antibacterial assay, *S. aureus* was adjusted to approximately 1 × 10^6^ CFU/mL and incubated with 0%, 2%, and 10% DH-EV-loaded hydrogels at 37 °C for 24 h, with bacterial suspension without hydrogel serving as the Control. The suspensions were serially diluted, plated on LB agar, and incubated at 37 °C for 24 h before colony counting. The bacterial survival rate was normalized to the Control group and calculated according to the following equation:

Bacterial survival rate = (*N*_t_/*N*_c_) × 100%

where N_t_ represents the colony number in the hydrogel-treated group and Nc represents the colony number in the Control group.

For the bacterial adhesion assay, the 0%, 2%, and 10% DH-EV-loaded hydrogels were incubated with 1 × 10^8^ CFU/mL *S. aureus* at 37 °C for 2 h. After incubation, the hydrogels were gently washed with PBS to remove non-adherent bacteria. The adhered bacteria were subsequently detached from the hydrogel surfaces by shaking, followed by serial dilution, plating on LB agar, and incubation at 37 °C for 24 h. The relative bacterial adhesion rate was normalized to the 0% DH-EVs hydrogel group and calculated according to the following equation:

Relative bacterial adhesion rate =(*N*_t_/*N*_0_) × 100%

where N_t_ represents the colony number of adhered bacteria recovered from the corresponding hydrogel group and N_0_ represents the colony number of adhered bacteria recovered from the DA-OSA-CMC-0%DH group.

#### 2.6.4. Anti-NETs Formation Assay

Neutrophils (5 × 10^5^ cells/well) were randomly assigned to five groups: control, PMA, and three dressing-extract groups. Extract groups received dressing extracts; others received equal-volume PBS. After 2 h, all noncontrol groups were exposed to PMA for 2 h to induce NETs.

Cells were washed, fixed with 4% paraformaldehyde (Biosharp, Beijing, China) for 15 min, blocked with 5% bovine serum albumin (Solarbio, Beijing, China) for 30 min, and incubated overnight at 4 °C with primary antibodies against Histone H3 (1:100) and MPO (1:100). Fluorescent secondary antibodies (both diluted 1:100) were applied for 2 h at room temperature in the dark. The samples were then mounted using antifade mounting medium with DAPI (Beyotime Biotechnology, Shanghai, China) and examined by inverted fluorescence microscopy (Ti2-U, Nikon, Tokyo, Japan). Supernatants were collected for NET measurement by ELISA.

### 2.7. In Vivo Experiments

#### 2.7.1. Establishment of Type 2 Diabetes Mouse Model

Five-week-old male C57BL/6J mice were purchased from Jinan Pengyue Experimental Animal Breeding Co., Ltd. (Jinan, China). The protocol was approved by the Ethics Committee of the Medical College of Qingdao University (Approval No. QDU-AEC-2025753). Procedures followed the Chinese Ministry of Science and Technology’s Regulations on Laboratory Animal Welfare, and mice received humane care.

Mice were randomized into four groups and fed a high-fat, high-sugar diet (Xiao Shu You Tai Biotechnology Co., Ltd., Beijing, China) for 4 weeks. Diabetes was induced by daily intraperitoneal STZ (25 mg/kg in pH 4.5 citrate buffer; Solarbio, Beijing, China) for 5 days in darkness. Fasting glucose was measured; >16.7 mmol/L indicated type 2 diabetes modeling.

#### 2.7.2. Wound Healing Model Establishment

Type 2 diabetic mice were anesthetized, and the dorsal hair was shaved. A 6 mm circular full-thickness wound was created on the back using a skin punch biopsy instrument, and the wound was infected with methicillin-resistant *Staphylococcus aureus* (MRSA) at a concentration of 10^8^ CFU•mL^−1^. The control group received no dressing treatment, while the DA-OSA-CMC-0%DH, DA-OSA-CMC-2%DH, and DA-OSA-CMC-10%DH groups received corresponding dressing treatments. Wound photographs were taken on days 0, 3, 6, 9, 12, 15, and 18 after treatment, and wound contraction rates were quantitatively analyzed using ImageJ software.

#### 2.7.3. Histological Staining

Paraffin-embedded tissue sections were deparaffinized and rehydrated following standard procedures, followed by hematoxylin and eosin (H&E) staining and Masson’s trichrome staining (Solarbio, Beijing, China). Immunofluorescence staining was performed on the remaining skin specimens.

#### 2.7.4. ELISA Detection

Mouse orbital blood was collected in tubes and allowed to clot naturally at room temperature for 10–20 min, then centrifuged at 2000–3000 rpm for approximately 20 min to collect the supernatant. The MPO-DNA kit was used to detect NET levels in serum.

#### 2.7.5. Immunofluorescence Staining of Tissue Sections

Immunofluorescence staining was performed on tissue sections using antibodies against CitH3 (1:100) and Ly6G (1:1000) to assess NET formation, and CD80 (1:100) and CD206 (1:100) (BioLegend, San Diego, CA, USA) to evaluate macrophage polarization. The sections were incubated with primary antibodies overnight at 4 °C, followed by the corresponding fluorescent secondary antibodies (both diluted 1:100) for 2 h at room temperature in the dark. DAPI was used for nuclear staining.

### 2.8. Statistical Analysis

Statistical analysis was performed using GraphPad Prism 10.1.2 and Origin 2025. Data are expressed as mean ± standard deviation. Comparisons between two groups were analyzed using Student’s t-test, and multiple-group comparisons were analyzed using one-way analysis of variance. Wound contraction rates measured repeatedly over time in the same animals were analyzed using a mixed-effects model with restricted maximum likelihood (REML), with treatment, time, and their interaction included as fixed effects and individual animals as a random effect. Dunnett’s multiple comparisons test was used to compare each treatment group with the model group at each time point. A *p*-value < 0.05 was considered statistically significant.

## 3. Results

### 3.1. Extraction and Characteristic Analysis of DH-EVs

**Figure 1:**
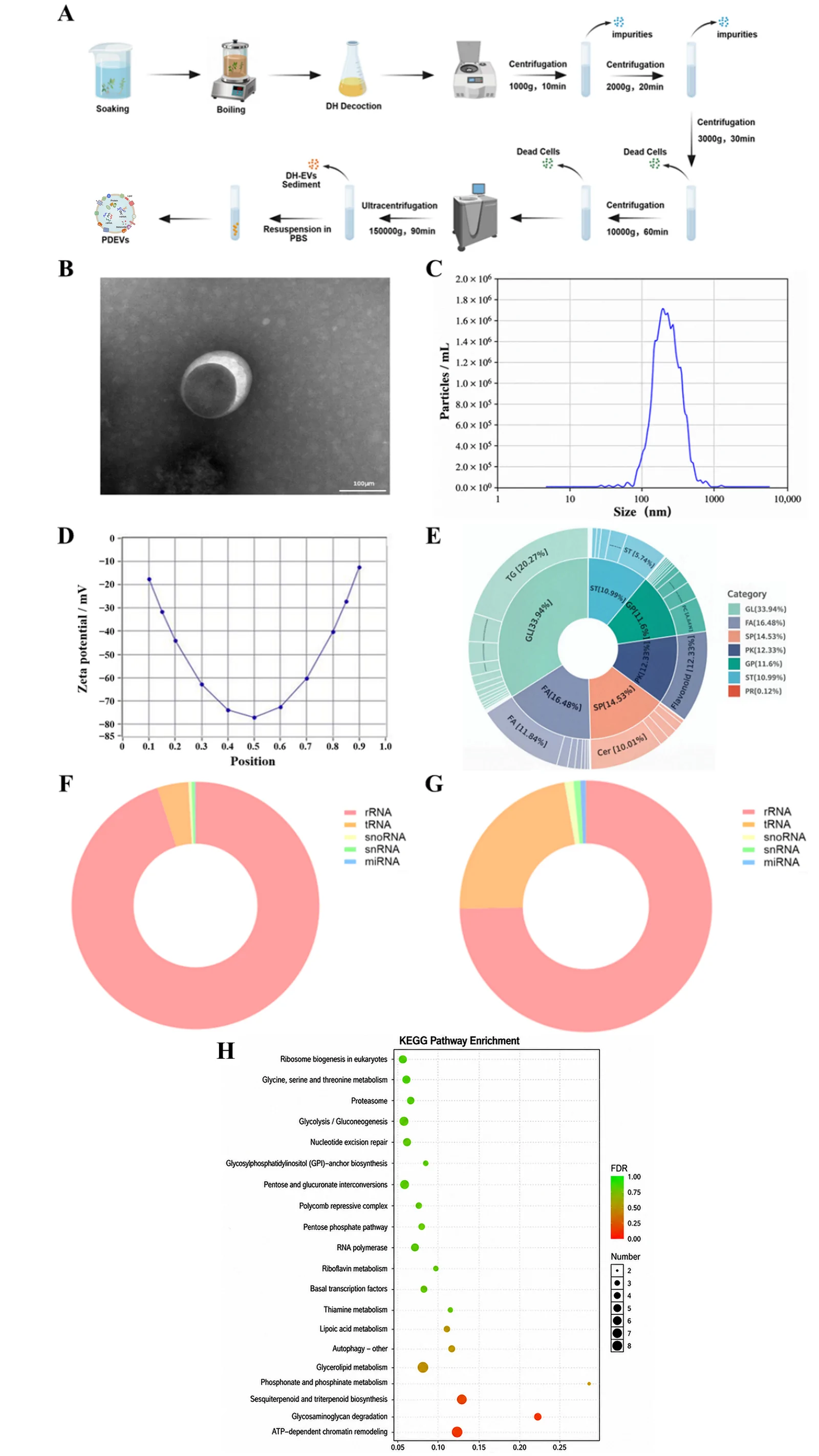
Extraction, characterization and sequencing results of DH-EVs. (**A**) Extraction process of DH-EVs. (**B**) Transmission electron microscopy image of DH-EVs showing morphology. (**C**) Particle size distribution and particle concentration of DH-EVs. (**D**) Zeta potential distribution of DH-EVs at different measurement positions (mean: −47.18 mV). (**E**) Untargeted lipid sequencing results of DH-EVs. (**F**) sRNA classification statistics of DH-EVs compared with the *Salvia miltiorrhiza* reference genome. (**G**) sRNA classification statistics of DH-EVs compared with the *Astragalus membranaceus* reference genome. (**H**) KEGG pathway enrichment analysis results of DH-EVs. The size of each circle represents the number of genes, and color indicates *p*-value.

Differential centrifugation is one of the commonly used methods for extracting plant-derived extracellular vesicles. Therefore, *Salvia miltiorrhiza* and *Astragalus membranaceus* were decocted at a 1:1 ratio, filtered, and DH-EVs were extracted by differential centrifugation. The overall isolation procedure is schematically illustrated in Figure 1A. To further characterize DH-EVs, BCA assay, nanoparticle tracking analysis (NTA), and transmission electron microscopy (TEM) were used to evaluate their concentration, particle size, zeta potential, and morphology. Results showed that the concentration of DH-EVs was approximately 1.07 × 10^2^ mg/mL, with a peak diameter of approximately 199 nm and an average zeta potential of approximately −47.18 mV. The vesicles exhibited intact bilayer membrane structures with a cup-shaped morphology, consistent with typical plant-derived extracellular vesicle characteristics (Figure 1B–D).

Lipids are important components of extracellular vesicles and play crucial roles in maintaining DH-EV structure and exerting their functions. Untargeted lipid sequencing results of DH-EVs (Figure 1E) revealed that DH-EVs were mainly composed of 33.94% glycerolipids (GL), 16.48% fatty acyls (FA), 14.53% sphingolipids (SP), 12.33% polyketides (PK), 11.6% glycerophospholipids (GP), and other lipids. These lipids are primarily involved in inflammatory regulation, apoptosis, and signal transduction processes. Subsequently, small RNA transcriptome sequencing analysis of DH-EVs was performed. Comparison of DH-EVs small RNAs with the reference genomes of *Salvia miltiorrhiza* and *Astragalus membranaceus* revealed that 0.06% (aligned to the *Salvia miltiorrhiza* reference genome) and 0.69% (aligned to the *Astragalus membranaceus* reference genome) of small RNAs were annotated as miRNAs (Figure 1F,G). Target gene prediction analysis of the screened microRNAs (miRNAs) revealed that these miRNAs were predominantly enriched in multiple pathways, including glycolysis/gluconeogenesis, pentose phosphate pathway, riboflavin metabolism, proteasome, autophagy, glycerolipid metabolism, glycosaminoglycan degradation, and ATP-dependent chromatin remodeling Figure 1H). Among these pathways, Glycolysis provides metabolic and energetic support for neutrophil activation and NET release [21]; the pentose phosphate pathway generates NADPH to sustain NADPH oxidase-dependent reactive oxygen species (ROS) production, and ROS act as vital drivers of canonical NETosis [22]. Flavin cofactors yielded via riboflavin metabolism, particularly FAD, also participate in redox reactions and ROS generation [23]. Autophagy and proteasome pathways may participate in the regulation of neutrophil activation and NET formation [24]. Moreover, the enrichment of chromatin regulatory pathways such as ATP-dependent chromatin remodeling and Polycomb repressive complex pathways suggests that chromatin opening, decondensation, or epigenetic modulation may contribute to NET release under inflammatory conditions [25]. Collectively, these enrichment data demonstrate that DH-EVs may modulate neutrophil activation and NET formation through a sequential cascade consisting of elevated glucose metabolism, NADPH/ROS production, autophagy/protein homeostasis regulation, and chromatin remodeling, thereby participating in the progression of inflammatory responses and tissue injury.

### 3.2. Preparation, Characterization, and Biocompatibility Evaluation of Multifunctional Hydrogel Loaded with DH-EVs

#### 3.2.1. FTIR Analysis of Multifunctional Hydrogel Loaded with DH-EVs

**Figure 2:**
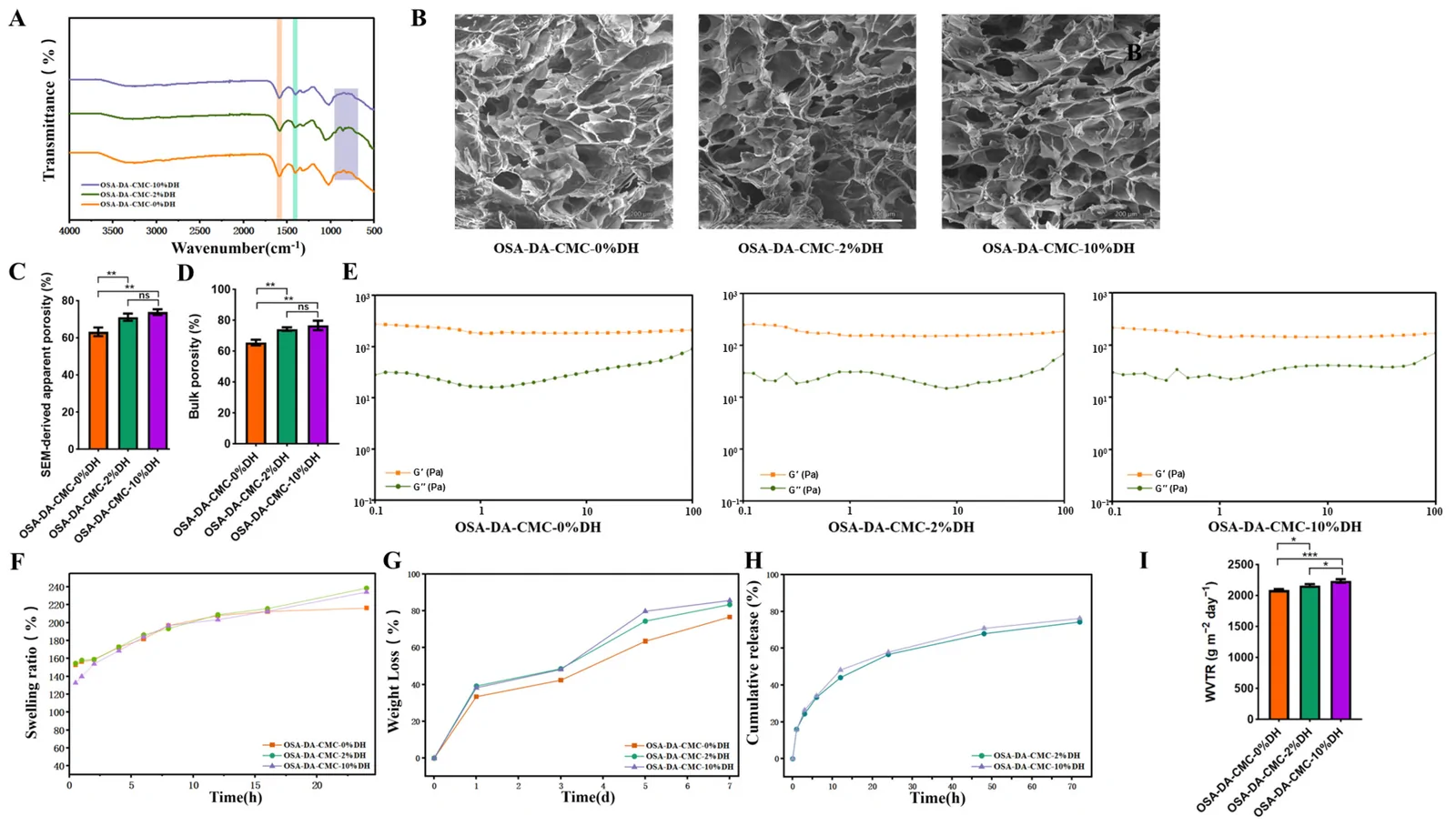
Characterization and performance evaluation of multifunctional hydrogel dressings loaded with DH-EVs. (**A**) FTIR spectral analysis of multifunctional hydrogel dressings loaded with DH-EVs. (**B**) SEM images of multifunctional hydrogel dressings loaded with DH-EVs. (**C**) SEM-derived apparent porosity quantified using ImageJ. (**D**) Bulk porosity determined by the ethanol displacement method. (**E**) Oscillatory strain sweep test results of multifunctional hydrogel dressings loaded with DH-EVs. (**F**) Swelling performance test results of multifunctional hydrogel dressings loaded with DH-EVs. (**G**) Degradation performance test results of multifunctional hydrogel dressings loaded with DH-EVs. (**H**) In vitro cumulative release profiles of DH-EVs from DA-OSA-CMC hydrogels. (**I**) Water vapor transmission rate (WVTR) of OSA-DA-CMC hydrogels containing different concentrations of DH-EVs. (* *p* < 0.05, ** *p* < 0.01, *** *p* < 0.001, ns indicates no statistical significance, *n* = 6).

Oxidized sodium alginate (OSA) and carboxymethyl chitosan (CMC) possess good biocompatibility and are commonly used as basic materials for hydrogel construction. In this study, FTIR was used to characterize the chemical features of OSA-DA-CMC hydrogels containing different amounts of DH-EVs (Figure 2A). The FTIR spectra showed similar characteristic absorption profiles among the three hydrogel groups. Distinct absorption bands were observed at approximately 1590 and 1400 cm^−1^, which were mainly associated with the stretching vibrations of carboxylate groups (–COO^−^), while the absorption around 1590 cm^−1^ may also contain overlapping contributions from imine (Schiff-base) and amide-related vibrations. In addition, the absorption features in the 950–650 cm^−1^ region may be associated with the aromatic ring structures of the dopamine moieties. Overall, these characteristic absorption signals were consistent with the expected chemical composition of the OSA-DA-CMC hydrogel. With increasing DH-EV content, no appreciable shifts in the major characteristic absorption bands were observed among the three hydrogel groups, suggesting that, within the scope of the FTIR analysis, DH-EV incorporation did not cause marked changes in the overall chemical characteristics of the OSA-DA-CMC hydrogel matrix.

#### 3.2.2. Morphological and Porosity Analysis of Multifunctional Hydrogel Loaded with DH-EVs

The microstructures of DA-OSA-CMC-0%DH, DA-OSA-CMC-2%DH, and DA-OSA-CMC-10%DH hydrogels were observed by SEM (Figure 2B). All three hydrogels exhibited typical interconnected porous network structures, indicating that the incorporation of DH-EVs did not obviously disrupt the overall porous framework of the hydrogel. Quantitative analysis of the SEM images showed that the apparent porosity increased from 63.23 ± 2.33% in the 0%DH group to 71.15 ± 1.97% and 73.88 ± 1.50% in the 2%DH and 10%DH groups, respectively (Figure 2C). Consistently, ethanol displacement measurements showed an increase in bulk porosity from 65.56 ± 1.84% to 74.22 ± 1.13% and 76.66 ± 3.07%, respectively (Figure 2D). For both measurements, the DH-EV-containing groups showed significantly higher porosity than the 0%DH group (*p* < 0.01), whereas no significant difference was observed between the 2%DH and 10%DH groups. Overall, these results indicate that the incorporation of DH-EVs increased hydrogel porosity while preserving the interconnected porous network. Such a porous structure may facilitate water absorption and fluid transport, thereby contributing to wound-exudate management and maintenance of a favorable moist wound environment.

#### 3.2.3. Mechanical Properties of Multifunctional Hydrogel Loaded with DH-EVs

Since diabetic wounds are often irregular in shape, dressings should possess suitable viscoelastic characteristics to facilitate conformity to the wound surface. Therefore, rheological tests were performed on the three dressings (Figure 2E) to determine their rheological behavior under increasing strain. Oscillatory strain sweep results showed that the storage modulus (G′) of all three hydrogels was higher than the loss modulus (G″) within the tested range, indicating predominantly elastic, solid-like rheological behavior. Additionally, OSA-DA-CMC-2%DH and OSA-DA-CMC-10%DH exhibited slightly lower G′ values than OSA-DA-CMC-0%DH under the same strain, while all three dressings maintained predominantly solid-like rheological characteristics within the tested strain range.

#### 3.2.4. Swelling and Degradation Properties of Multifunctional Hydrogel Loaded with DH-EVs

Hydrogel exudate absorption was further evaluated. Swelling tests (Figure 2F) showed that all three hydrogels swelled progressively and reached equilibrium within 24 h. DH-EVs slightly enhanced swelling, with OSA-DA-CMC-10%DH showing the highest equilibrium swelling ratio, consistent with its finer, more uniform porous structure, which improved water absorption and maintained a moist healing environment.

The degradation behavior of the hydrogels in PBS was also evaluated. Degradation tests (Figure 2G) showed time-dependent mass loss in all hydrogels, with degradation accelerating as DH-EVs content increased. OSA-DA-CMC-10%DH degraded fastest and had the greatest mass loss on day 7. These results suggest that DH-EVs content may affect the swelling and degradation behavior of the hydrogels in PBS, with higher DH-EVs content resulting in a higher swelling ratio and a faster degradation rate.

#### 3.2.5. In Vitro Release Behavior of DH-EVs from Multifunctional Hydrogel

The release behavior of DH-EVs from DA-OSA-CMC hydrogels was evaluated in PBS over 72 h (Figure 2H). Both DA-OSA-CMC-2%DH and DA-OSA-CMC-10%DH exhibited sustained release profiles, characterized by relatively rapid release at the initial stage followed by a gradual decrease in release rate. The cumulative release increased continuously over time and reached approximately 74–76% at 72 h. Although the DA-OSA-CMC-10%DH group showed slightly higher cumulative release than the DA-OSA-CMC-2%DH group, the overall release trends were similar. These results indicate that the DA-OSA-CMC hydrogel could effectively retain DH-EVs and provide sustained release over an extended period.

#### 3.2.6. Water Vapor Transmission Rate of Multifunctional Hydrogel Loaded with DH-EVs

Water vapor transmission rate (WVTR) analysis of the three hydrogels (Figure 2I) showed that the WVTR values of OSA-DA-CMC-0%DH, OSA-DA-CMC-2%DH, and OSA-DA-CMC-10%DH were 2087 ± 15, 2162 ± 21, and 2235 ± 28 g m^−2^ day^−1^, respectively. WVTR increased with increasing DH-EVs content, and significant differences were observed among the three groups. This trend was consistent with the enhanced swelling capacity of the hydrogels after DH-EVs incorporation, suggesting improved moisture transport through the hydrogel matrix. The enhanced water vapor permeability may facilitate moisture exchange and contribute to maintaining an appropriate moist microenvironment for wound healing.

#### 3.2.7. Cytotoxicity and Biocompatibility of Multifunctional Hydrogel Loaded with DH-EVs

**Figure 3:**
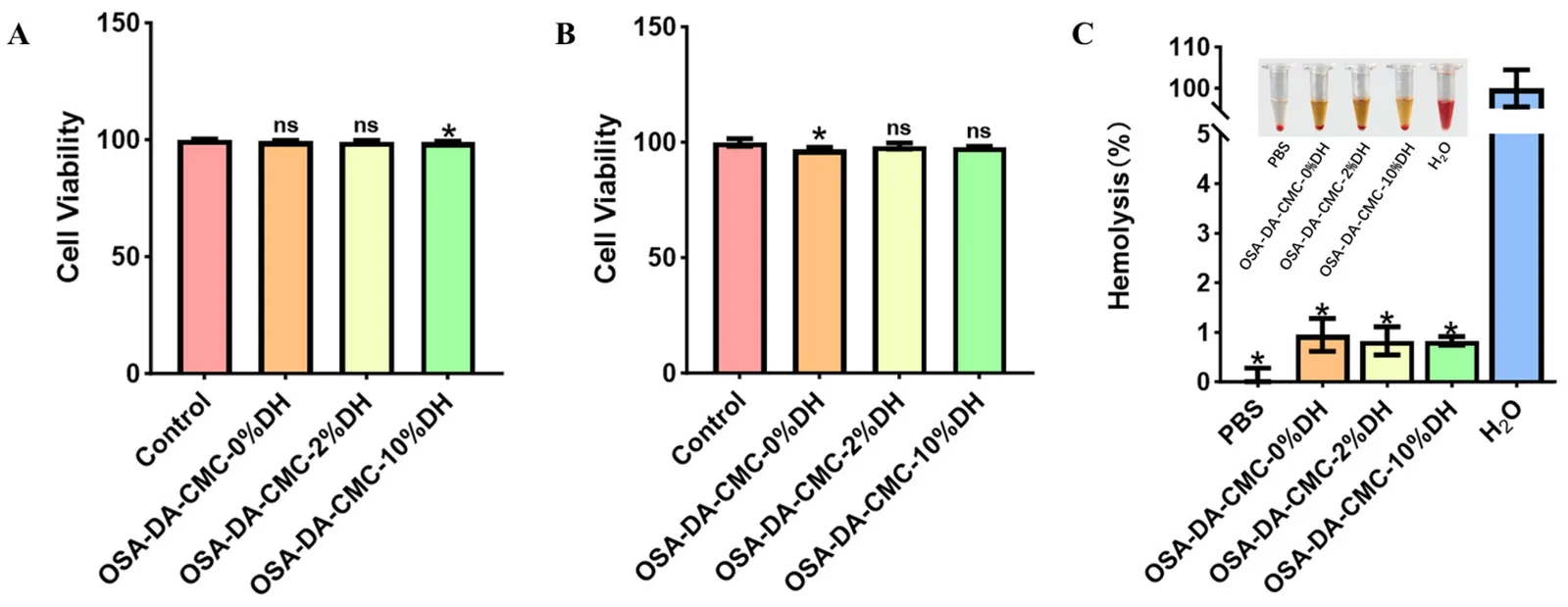
Cytotoxicity and biocompatibility of multifunctional hydrogel dressings loaded with DH-EVs. (**A**) Fibroblast cell viability test results of multifunctional hydrogel dressings loaded with DH-EVs (* *p* < 0.05 vs. control, ns indicates no statistical significance vs. control, *n* = 6). (**B**) Neutrophil cell viability test results of multifunctional hydrogel dressings loaded with DH-EVs (* *p* < 0.05 vs. control, ns indicates no statistical significance vs. control, *n* = 6). (**C**) Hemolysis assay images and absorbance analysis of multifunctional hydrogel dressings loaded with DH-EVs (* *p* < 0.05 vs. H_2_O, *n* = 6).

Cytotoxicity and biocompatibility are primary criteria determining whether medical dressings can be applied clinically. CCK-8 assays were performed to test the viability of fibroblasts and neutrophils cultured with extracts of OSA-DA-CMC hydrogels containing different DH-EV contents. After 24 h of culture, fibroblast viability in all hydrogel extract groups remained above 98% (Figure 3A), indicating good cytocompatibility and no obvious cytotoxicity. For neutrophils, results (Figure 3B) showed that all hydrogel extract groups exhibited good biocompatibility, with neutrophil viability above 90% and no obvious cytotoxicity. The OSA-DA-CMC-2%DH and OSA-DA-CMC-10%DH groups showed no statistically significant differences compared with the control group, indicating that DH-EVs incorporation maintained good cytocompatibility. Overall, all three hydrogels had no negative effects on the viability of fibroblasts or neutrophils and exhibited good in vitro biosafety, serving as the basis for subsequent experiments.

To meet biosafety requirements for biomaterials, the hemolysis rate of clinical dressings should be controlled below 5% according to ISO/TR 7406 standards [26]. Hemolysis assay results (Figure 3C) showed that the H_2_O (positive control) group exhibited significant hemolysis, while the PBS group, OSA-DA-CMC-0%DH group, OSA-DA-CMC-2%DH group, and OSA-DA-CMC-10%DH group showed no hemolysis. Subsequent absorbance analysis using a microplate reader revealed that the hemolysis rates of all three hydrogel extract groups were far below the 5% safety threshold.

### 3.3. Antibacterial and Anti-Adhesive Properties of DH-EV-Loaded Multifunctional Hydrogels

**Figure 4:**
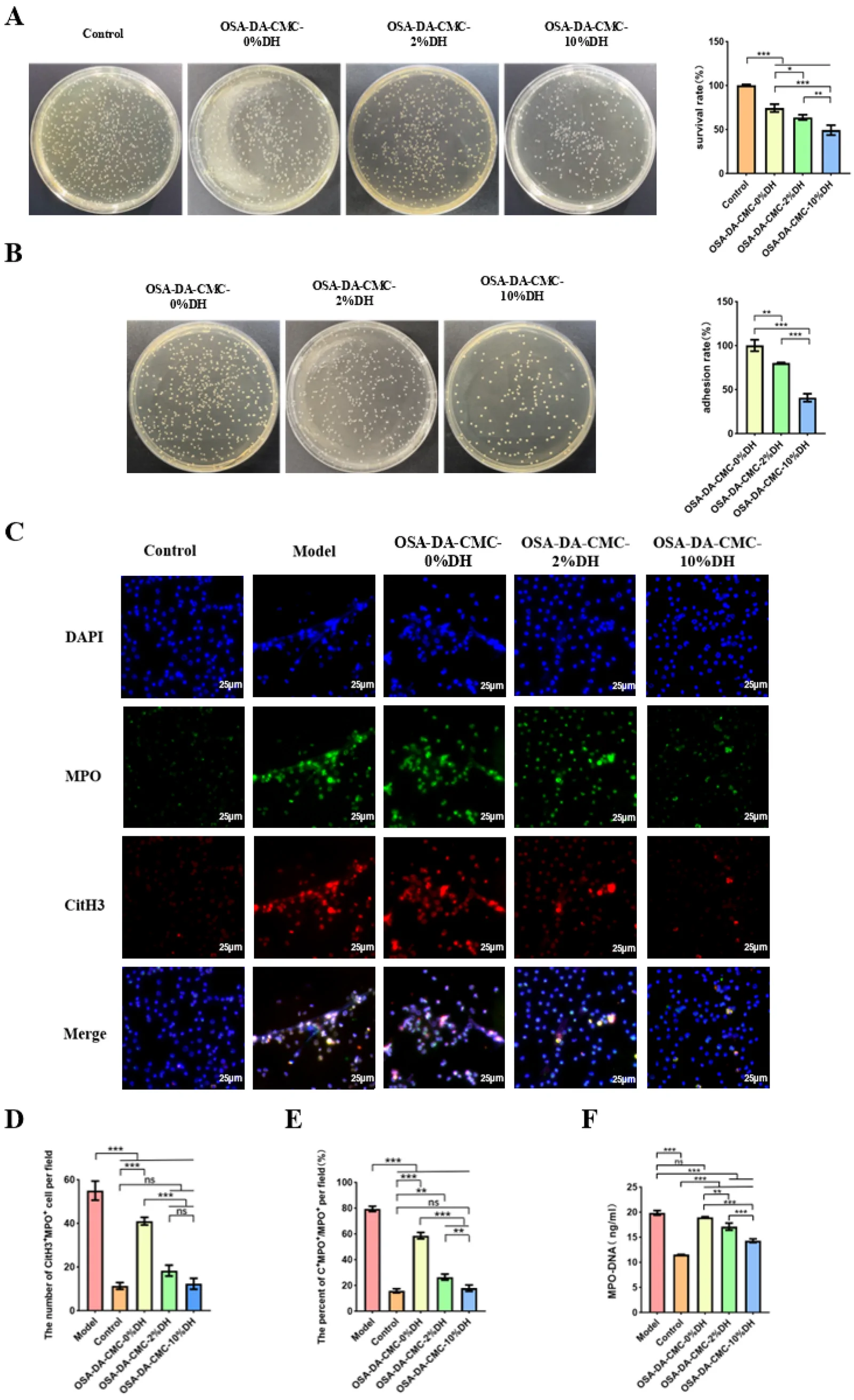
Evaluation of the Antibacterial, Anti-adhesive, and Anti-NETs Formation Properties of DH-EVs-Loaded Multifunctional Hydrogel Dressings. (**A**) Antibacterial activity of *S. aureus* after incubation with three DH-EVs-loaded hydrogel dressings and quantitative analysis of bacterial survival rates. (**B**) Bacterial adhesion of *S. aureus* on three DH-EVs-loaded hydrogel dressings and quantitative analysis of bacterial adhesion rates. (**C**) Immunofluorescence staining of NETs in neutrophils after incubation with three hydrogel extracts: MPO (neutrophil marker, green) and CitH3 (NET marker, red), scale bar: 25 μm. (**D**) Quantification of CitH3^+^MPO^+^ cells in immunofluorescence staining. (**E**) Percentage of CitH3^+^MPO^+^/MPO^+^ cells in immunofluorescence staining. (**F**) ELISA quantitative detection of MPO-DNA (NET marker) in supernatants of neutrophils incubated with three hydrogel extracts. *n* = 6, * *p* < 0.05, ** *p* < 0.01, *** *p* < 0.001, ns indicates no statistical significance.

The hydrogels exhibited different degrees of antibacterial activity against *Staphylococcus aureus* (*S. aureus*). Compared with the Control group, hydrogel treatment markedly reduced the number of viable bacterial colonies. Bacterial survival showed a further decreasing trend with increasing DH-EVs loading, with the OSA-DA-CMC-10%DH group exhibiting a more pronounced inhibitory effect on bacterial growth. Quantitative analysis further confirmed this trend, with statistically significant differences observed between selected groups (Figure 4A). These results indicate that Multifunctional Hydrogel Loaded with DH-EVs effectively reduces the survival of *S. aureus*, with higher DH-EVs loading being associated with a more pronounced antibacterial effect.

The initial adhesion of *S. aureus* to different hydrogel surfaces was further evaluated. After incubation, the number of recoverable viable bacteria differed markedly among the hydrogel groups and progressively decreased with increasing DH-EVs loading. Compared with the OSA-DA-CMC-0%DH group, fewer adherent bacteria were recovered from the DH-EV-loaded hydrogels, with the OSA-DA-CMC-10%DH group exhibiting the lowest level of bacterial adhesion. Quantitative analysis of relative bacterial adhesion further supported this trend, with significant differences observed among the groups (Figure 4B). These findings indicate that the initial adhesion of *S. aureus* to the hydrogel surface decreases with increasing DH-EVs loading, demonstrating favorable anti-adhesive properties.

Overall, the DH-EVs-loaded multifunctional hydrogels reduced both the survival of *S. aureus* and its initial adhesion to the material surface, with both parameters showing a decreasing trend as DH-EVs loading increased. Among the formulations tested, the OSA-DA-CMC-10%DH group exhibited the lowest bacterial survival and adhesion levels, suggesting superior antibacterial and anti-adhesive properties.

### 3.4. Anti-NETs Formation Capacity of Multifunctional Hydrogel Loaded with DH-EVs

Excessive NET formation is a key factor leading to impaired diabetic wound healing. It not only prolongs the inflammatory phase by affecting macrophage polarization but also impairs endothelial cell activity and hinders angiogenesis [27,28]. Therefore, to evaluate the role of multifunctional hydrogel loaded with *Salvia miltiorrhiza*–*Astragalus membranaceus* extracellular vesicles in inhibiting neutrophil NET formation, immunofluorescence staining for citrullinated histone H3 (CitH3) and myeloperoxidase (MPO) was performed. The results show that, compared with the control group, CitH3 and MPO signal enrichment was observed in the PMA group and all extract-treated groups (Figure 4C). The PMA group exhibited obvious chromatin decondensation and neutrophil disruption, while the three hydrogel extracts inhibited neutrophil NET formation to varying degrees, with the inhibitory effect gradually strengthening with increasing DH-EVs content (Figure 4D,E). These findings were consistent with the quantitative MPO-DNA detection results in cell culture supernatants (Figure 4F).

### 3.5. Multifunctional Hydrogel Loaded with DH-EVs Promotes Wound Healing in Type 2 Diabetic Mice with Infected Wounds

**Figure 5:**
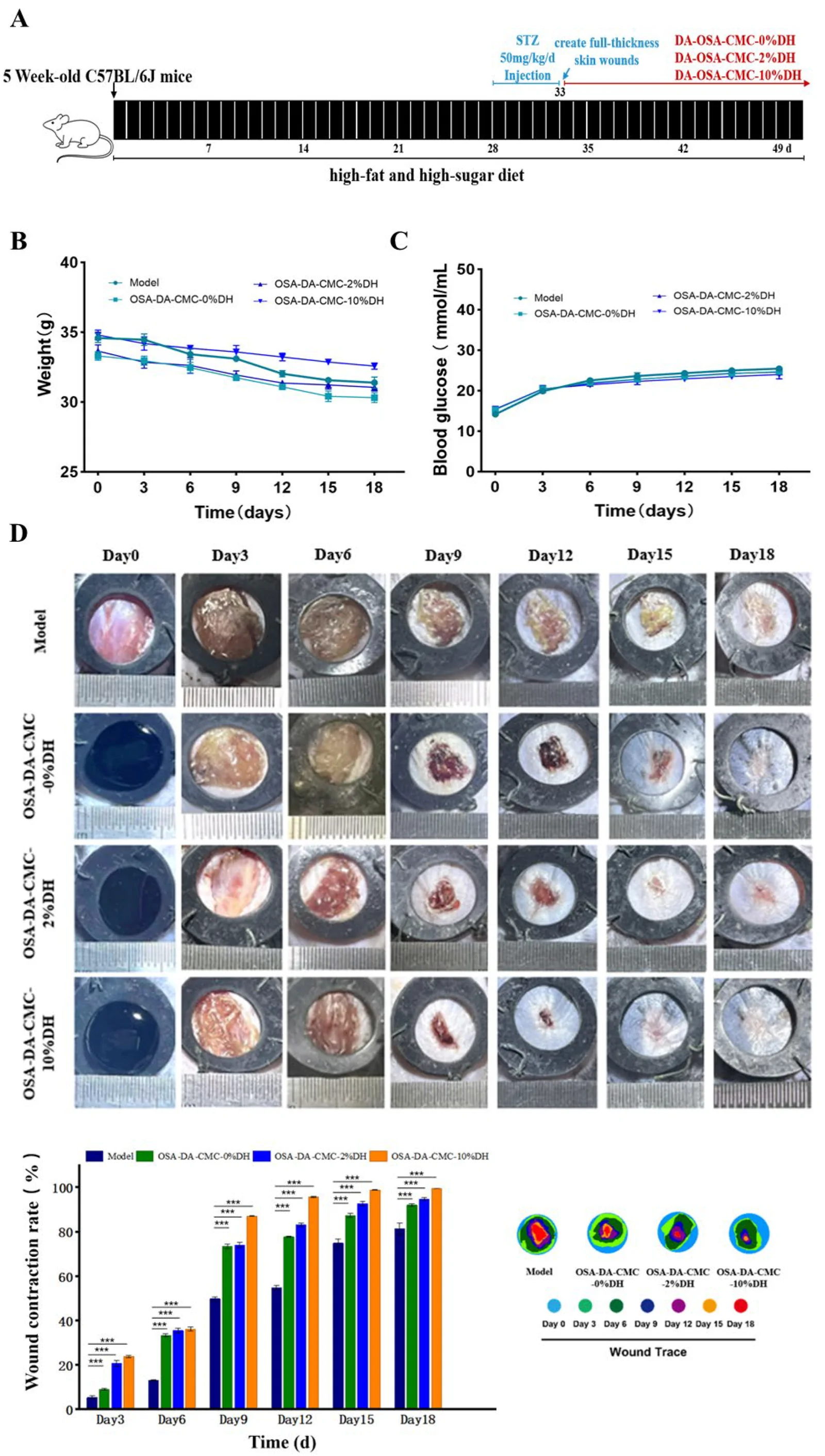
Multifunctional hydrogel dressing loaded with DH-EVs promotes wound healing in diabetic mice with bacterial infection. (**A**) Animal experiment process. (**B**) Body weight change curve of mice after modeling. (**C**) Blood glucose change curve of mice after modeling. (**D**) Representative wound images, wound traces, and wound contraction rates at the indicated time points. Data are mean ± SD (*n* = 6). Mixed-effects model (REML) followed by Dunnett’s multiple comparisons test; *** *p* < 0.001 vs. Model group.

The overall modeling procedure is schematically illustrated in Figure 5A. After 5 weeks of a high-fat, high-sugar diet and STZ injections, blood glucose and body weight were monitored in C57BL/6J mice (Figure 5B,C). All four groups had blood glucose levels ≥ 16.7 mmol/L, together with weight loss and polydipsia/polyuria, confirming successful modeling.

Wounds were photographed on days 0, 3, 6, 9, 12, 15, and 18 (Figure 5D). On day 6, the Model group showed purulence, the OSA-DA-CMC-0% group had slight exudation, and the OSA-DA-CMC-2%DH and OSA-DA-CMC-10%DH groups showed wound contraction. By day 9, wound areas had decreased in all groups. However, the Model group healed slowly with persistent purulent exudate, whereas all hydrogel-treated groups showed faster healing, indicating that the dressings promoted the closure of bacterially infected diabetic wounds. By day 18, wounds were essentially healed with new hair growth; the OSA-DA-CMC-10%DH group showed the best outcome, with a gross appearance closest to normal skin.

Quantitative analysis showed that wound contraction rates increased over time in all groups. Mixed-effects analysis revealed significant effects of time, treatment, and their interaction (all *p* < 0.001). On days 3, 6, 9, 12, 15, and 18, OSA-DA-CMC-10%DH achieved significantly higher wound contraction rates than the Model group and showed the highest values among the hydrogel-treated groups.

### 3.6. Histological Evaluation

**Figure 6:**
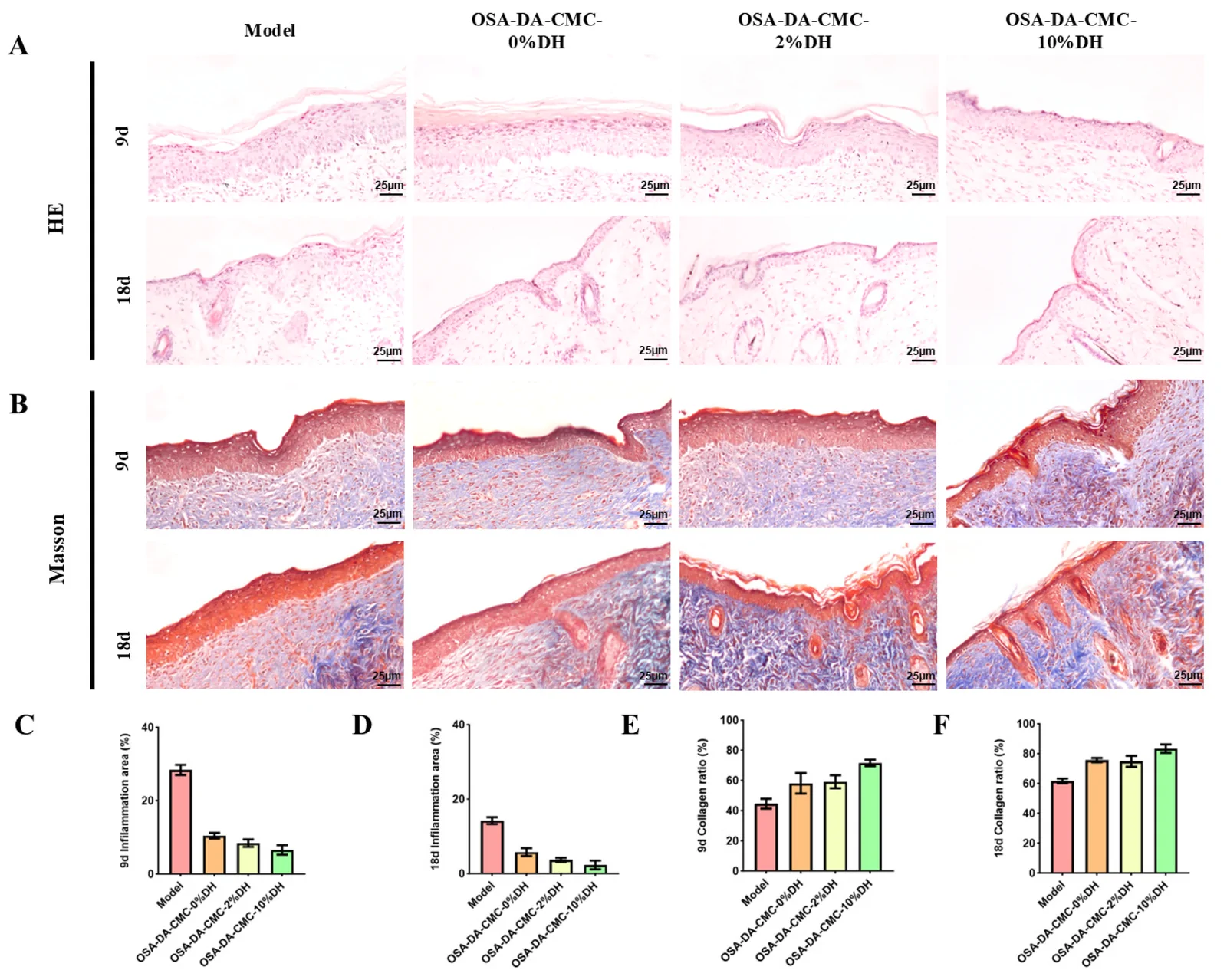
Histological evaluation of wound healing in diabetic mice with bacterial infection. (**A**) H&E staining images of wounds on days 9 and 18. (**B**) Masson’s trichrome staining images of wounds on days 9 and 18. Scale bar: 25 μm. (**C**–**F**) Quantitative results of inflammatory infiltration and collagen deposition in skin tissues at Day 9 and Day 18.

To further evaluate the promoting effect of multifunctional hydrogel loaded with DH-EVs on wound healing in type 2 diabetic mice, wound and surrounding skin tissues were collected on days 9 and 18 for H&E staining and Masson’s trichrome staining. At day 9 (Figure 6A), varying degrees of inflammatory cell infiltration and tissue defects were observed in all groups, with obvious healing trends after 18 days of treatment. Notably, the OSA-DA-CMC-10%DH group exhibited milder inflammatory responses and more favorable healing trends at both day 9 and day 18. Masson staining results (Figure 6B) further reflected collagen deposition in wound tissues of each group. The Model group showed sparse and disorganized collagen fiber deposition with persistent inflammatory cell infiltration; in contrast, the multifunctional hydrogel groups with different DH-EVs concentrations exhibited more abundant and orderly collagen fibers, along with the appearance of newly formed hair follicles and sebaceous glands, with the promoting effect increasing with DH-EVs concentration. Quantitative analysis of inflammatory infiltration and collagen deposition (Figure 6C–F) further confirmed these histological observations, showing reduced inflammatory infiltration and increased collagen deposition in the multifunctional hydrogel groups at both day 9 and day 18, with the OSA-DA-CMC-10%DH group exhibiting the most pronounced effects. These results collectively demonstrate that the three prepared multifunctional hydrogel dressings all played significant roles in reducing wound inflammatory responses and enhancing collagen deposition.

### 3.7. In Vivo Anti-NETs Formation Capacity Evaluation

**Figure 7:**
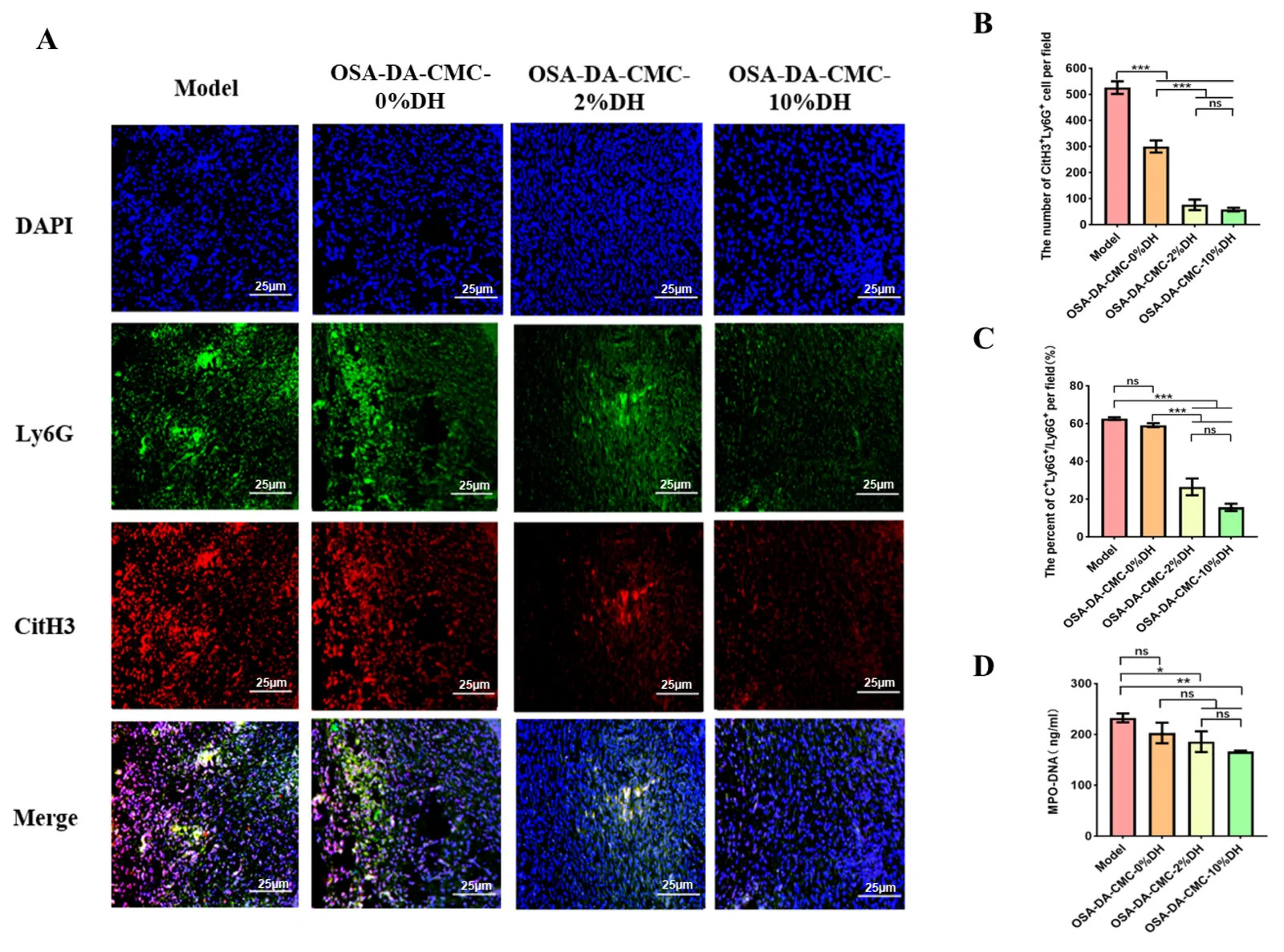
In vivo anti-NETs formation and anti-inflammatory capacity of multifunctional hydrogel dressings loaded with DH-EVs. (**A**) Immunofluorescence detection of wound NETs: Ly6G (neutrophil marker, green) and CitH3 (NET marker, red), scale bar: 25 μm. (**B**) Quantification of CitH3^+^Ly6G^+^ cells in immunofluorescence staining. (**C**) Percentage of CitH3^+^Ly6G^+^/Ly6G^+^ cells in immunofluorescence staining. (**D**) ELISA quantitative detection of MPO-DNA (NET marker) in serum. *n* = 6, * *p* < 0.05, ** *p* < 0.01, *** *p* < 0.001, ns indicates no statistical significance.

To evaluate the in vivo anti-NET effect of DH-EVs-loaded multifunctional hydrogel dressings, serum MPO-DNA complex levels were measured by ELISA (Figure 7D). The Model group showed the highest MPO-DNA level, whereas all three hydrogel-treated groups showed reduced serum NET levels. The reduction was DH-EVs dose-dependent, with higher DH-EVs contents corresponding to lower MPO-DNA levels.

Ly6G, a neutrophil marker, and CitH3 indicate local neutrophil infiltration and NET release at wound sites. Dual immunofluorescence staining showed that the Model group had the strongest Ly6G and CitH3 signals, both highly co-localized with DAPI-stained chromatin, indicating extensive neutrophil infiltration and NET formation (Figure 7A–C). Among treated groups, OSA-DA-CMC-0%DH retained relatively strong Ly6G and CitH3 signals, indicating limited inhibition of neutrophil infiltration and NET formation. OSA-DA-CMC-2%DH markedly reduced CitH3 intensity, although neutrophil infiltration remained substantial. In OSA-DA-CMC-10%DH, both Ly6G and CitH3 fluorescence intensities were significantly reduced, with almost no co-localization observed. These results indicate that DH-EVs-loaded hydrogels reduced neutrophil infiltration and NET formation, with stronger effects at higher DH-EVs contents. The immunofluorescence findings were consistent with serum MPO-DNA quantification.

### 3.8. In Vivo Anti-Inflammatory Capacity Evaluation

**Figure 8:**
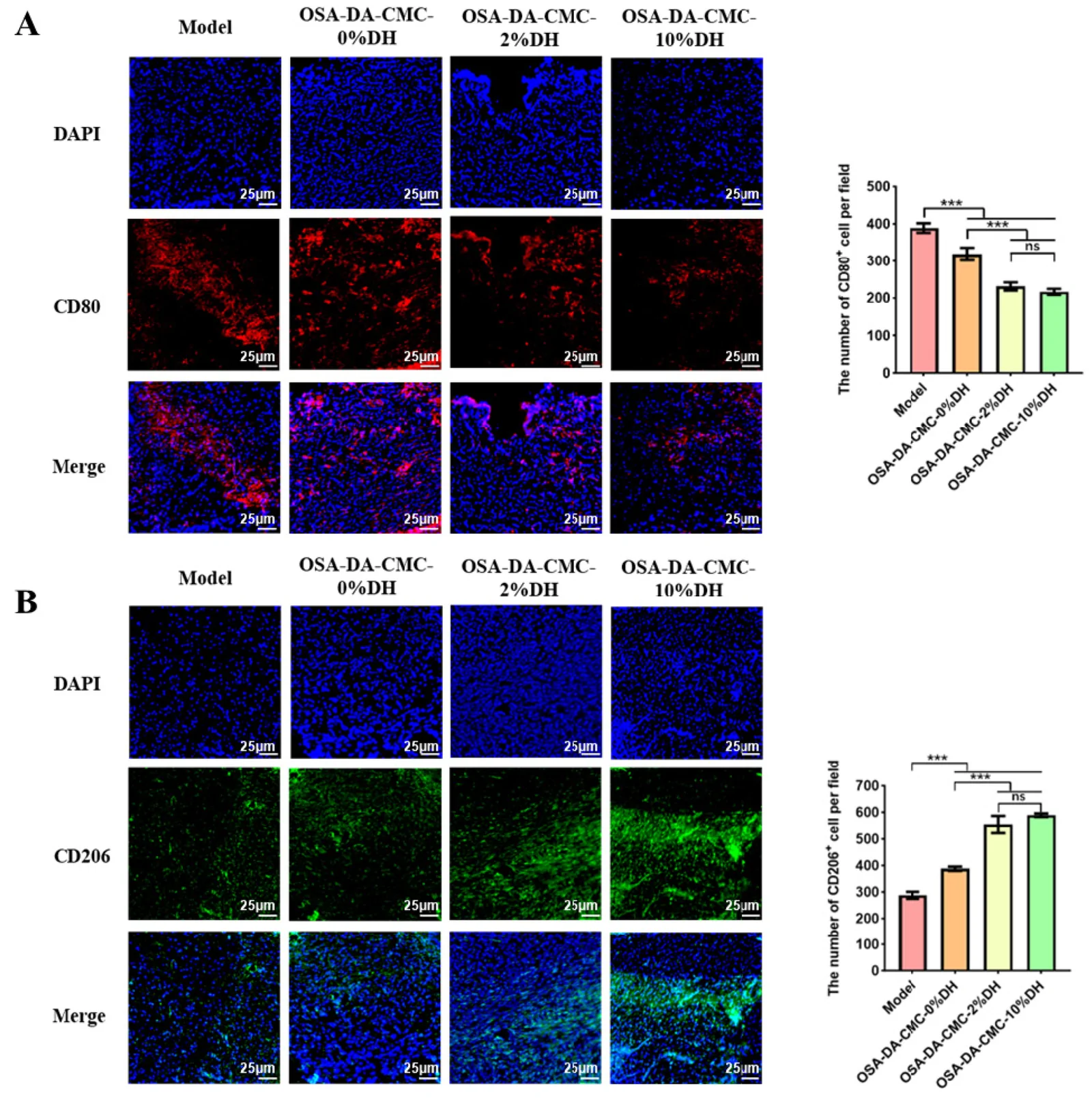
In vivo anti-inflammatory capacity evaluation of multifunctional hydrogel dressings loaded with DH-EVs. (A,B) Immunofluorescence staining images and quantification of wound macrophages: CD80 (M1 marker, red) and CD206 (M2 marker, red), scale bar: 25 μm. *n* = 6, *** *p* < 0.001, ns indicates no statistical significance.

To further verify whether the multifunctional hydrogel could reduce wound inflammatory responses by inhibiting NETs, immunofluorescence staining of CD80 (M1 macrophage marker) and CD206 (M2 macrophage marker) was performed on wound tissues. Results (Figure 8A,B) showed that the Model group exhibited significantly enhanced CD80 fluorescence signal and weak CD206 signal. The OSA-DA-CMC-10%DH group showed markedly downregulated CD80 expression and significantly upregulated CD206 expression, suggesting macrophage polarization from the pro-inflammatory M1 phenotype toward the anti-inflammatory M2 phenotype. Notably, the expression changes in CD80/CD206 were consistent with the trends of Ly6G/CitH3 reflecting neutrophil infiltration and NET formation. This result suggests that multifunctional hydrogels loaded with DH-EVs may reduce wound inflammatory responses by inhibiting NET formation.

## 4. Discussion

Healing of bacteria-infected diabetic wounds is impaired by hyperglycemia, oxidative stress, and immune microenvironment imbalance. Among these factors, persistent chronic inflammation caused by immune cell dysfunction is central to delayed wound repair [29]. This study shows that multifunctional hydrogel dressings loaded with DH-EVs alleviate local inflammation by suppressing NET formation, thereby accelerating the healing of bacteria-infected diabetic wounds.

Excessive NET formation aggravates inflammation in diseases such as atherosclerosis, lupus, and cirrhosis through NF-κB signaling and pro-inflammatory inflammasome activation [30]. In bacteria-infected diabetic wounds, bacterial biofilms impair neutrophil phagocytosis and induce excessive NET production, sustaining local inflammation [31]. Previous studies have shown that inhibiting NET generation can regulate macrophage polarization and reduce inflammation in diabetic wound models [28,32]. Consistently, our results showed that reduced NET formation decreased the proportion of pro-inflammatory M1 macrophages in wound tissues.

Current strategies for NET regulation mainly involve targeted degradation or inhibition of NET formation [4,33]. *Salvia miltiorrhiza* and *Astragalus membranaceus* are traditional Chinese medicines with multi-target effects, including inhibition of NET formation, antibacterial activity, and hypoglycemic potential. Their extracellular vesicles, DH-EVs, retain these therapeutic properties while improving stability, metabolic clearance, tissue efficacy, and sustained release.

However, direct injection of DH-EVs cannot maintain effective local concentrations. Therefore, DH-EVs were incorporated into oxidized sodium alginate hydrogel for local controlled release and improved bioavailability. Dopamine grafting further enhanced dressing adhesion [34]. The results showed that DH-EVs-loaded multifunctional hydrogel dressings inhibited NET formation in a content-dependent manner. Dressings without DH-EVs also promoted wound healing, but showed weaker NET-inhibitory effects. This may be related to the intrinsic anti-inflammatory, exudate-absorbing, pro-angiogenic properties of oxidized sodium alginate-based hydrogels and the moist healing environment they provide [35].

In summary, the DH-EVs-loaded multifunctional hydrogel dressing inhibited NET formation, reduced wound inflammation, and synergistically promoted the healing of bacteria-infected diabetic wounds through antibacterial and exudate-absorbing effects. However, the mechanism by which DH-EVs inhibit NET formation remains unclear. Several limitations of this study should be acknowledged. First, the absence of a free DH-EVs treatment group limited our ability to directly distinguish the intrinsic therapeutic effects of DH-EVs from those associated with hydrogel-mediated delivery. Second, although the OSA-DA/CMC ratio was selected based on previous studies and formulation considerations, different polymer compositions were not systematically optimized, which may limit a comprehensive understanding of the relationship between hydrogel composition and biological performance. Third, the degradation behavior of the hydrogel was evaluated only in PBS, without further assessment under enzyme-mediated conditions, and thus its enzyme-specific degradation kinetics and long-term biodegradability remain to be systematically investigated. Finally, the pro-angiogenic effects of this hydrogel were not evaluated, and its efficacy was verified only in cellular and animal models. Further studies are needed to evaluate its safety and therapeutic effects in different wound types and stages.

## 5. Conclusions

In this study, multifunctional hydrogel dressings loaded with different concentrations of DH-EVs were designed and developed. It was confirmed that DH-EV incorporation did not affect the morphology, structure, mechanical properties, or biocompatibility of the dressings. Meanwhile, the multifunctional hydrogel dressing containing 10% DH-EVs significantly inhibited NET formation in wounds, thereby promoting macrophage polarization toward the M2 phenotype and reducing wound inflammatory responses. In conclusion, the multifunctional hydrogel dressing loaded with DH-EVs effectively inhibits NET formation and synergistically promotes healing of bacteria-infected diabetic wounds through antibacterial and pro-exudate absorption functions.

## Data Availability

The data presented in this study are available from the corresponding author upon reasonable request.
